# Symptom Clusters and Functional Impairment in Individuals Treated for Lyme Borreliosis

**DOI:** 10.3389/fmed.2020.00464

**Published:** 2020-08-21

**Authors:** Nevena Zubcevik, Charlotte Mao, Qing Mei Wang, Eliezer L. Bose, Rose Nadlyne Octavien, David Crandell, Lisa J. Wood

**Affiliations:** ^1^Department of Physical Medicine and Rehabilitation, Harvard Medical School, Boston, MA, United States; ^2^The Dean Center for Tickborne Illness, Spaulding Research Institute, Spaulding Rehabilitation Hospital, Boston, MA, United States; ^3^Invisible International, Cambridge, MA, United States; ^4^Massachusetts General Hospital, Department of Pediatric Infectious Diseases, Boston, MA, United States; ^5^Stroke Biological Recovery Laboratory, Spaulding Research Institute, Spaulding Rehabilitation Hospital, Boston, MA, United States; ^6^Massachusetts General Hospital, Institute for Health Professions, School of Nursing, Charlestown, MA, United States; ^7^William F. Connell School of Nursing, Boston College, Chestnut Hill, MA, United States

**Keywords:** lyme borreliosis, symptom cluster, disability, fatigue, neurocognitive impairment

## Abstract

**Context:** Persistent fatigue, pain, and neurocognitive impairment are common in individuals following treatment for Lyme borreliosis (LB). Poor sleep, depression, visual disturbance, and sensory neuropathies have also been reported. The cause of these symptoms is unclear, and widely accepted effective treatment strategies are lacking.

**Objectives:** To identify symptom clusters in people with persistent symptoms previously treated for LB and to examine the relationship between symptom severity and perceived disability.

**Methods:** This was a retrospective chart review of individuals with a history of treatment of LB referred to The Dean Center for Tick-Borne Illness at Spaulding Rehabilitation Hospital between 2015 and 2018 (*n* = 270) because of persistent symptoms. Symptoms and functional impairment were collected using the General Symptom Questionnaire-30 (GSQ-30), and the Sheehan Disability Scale. Clinical tests were conducted to evaluate for tick-borne co-infections and to rule out medical disorders that could mimic LB symptomatology. Exploratory factor analysis was performed to identify symptom clusters.

**Results:** Five symptom clusters were identified. Each cluster was assigned a name to reflect the possible underlying etiology and was based on the majority of the symptoms in the cluster: the neuropathy symptom cluster, sleep-fatigue symptom cluster, migraine symptom cluster, cognitive symptom cluster, and mood symptom cluster. Symptom severity for each symptom cluster was positively associated with global functional impairment (p < 0.001).

**Conclusion:** Identifying the interrelationship between symptoms in post-treatment LB in a cluster can aid in the identification of the etiological basis of these symptoms and could lead to more effective symptom management strategies.

**Key Message:** This article describes symptom clusters in individuals with a history of Lyme borreliosis. Five clusters were identified: sleep-fatigue, neuropathy, migraine-like, cognition, and mood clusters. Identifying the interrelationship between symptoms in each of the identified clusters could aid in more effective symptom management through identifying triggering symptoms or an underlying etiology.

## Introduction

Lyme borreliosis (LB) is caused by various tick-borne genospecies of the spirochete bacteria *Borrelia burgdorferi sensu lato* (1, 2) and is a multisystem, multi-stage disease. LB is the most common vector-borne illness in the US, and the number of cases has increased steadily over the last 25 years (3). Transmission of the *B. burgdorferi* spirochete from infected ticks to its human host begins with the translocation of *B. burgdorferi* from the gut to the salivary glands of infected ticks while feeding on its human host (4). At the time of initial presentation of LB, *erythema migrans* (commonly described as a “bullseye” rash or an expanding, homogeneously red rash), is observed in <60% of infected patients within 7–10 days at the site of the tick bite (5–8). The rash usually resolves within weeks, even in the absence of antibacterial therapy. Additional signs of acute disseminated LB include fever, fatigue, muscle and joint pain, headache, and lymphadenopathy (5). Notably, other tickborne infections such as Anaplasma and Ehrlichia can manifest with similar flu-like symptoms and when co-existing with *B.burgdorferi*, increase the severity of the presentation. If *erythema migrans* is absent at the onset of infection and the flu-like symptoms are presumed to be related to non-specific viral infection (9), LB can go undiagnosed and untreated for weeks, months, or even years. Importantly, the spirochete can enter the bloodstream and disseminate, often affecting the heart, joints, and nervous system (5). Lyme neuroborreliosis (LNB) is reported to occur in 10–15% of LB patients although this may be an underestimate, as *Borrelia burgdorferi* has been shown to disseminate to the central nervous system (CNS) very early in the course of acute disseminated infection with minimal if any clinical evidence of CNS involvement (10). Furthermore, a latent neuroborreliosis can exist for quite some time without significant symptoms, then present with late CNS involvement many months to years after initial infection and less characteristic symptoms (11). Symptoms may include facial paralysis and other cranial neuropathies, headache, neck stiffness, fatigue, paresthesias, meningeal signs, depression, anxiety disorders, peripheral nervous system problems, encephalitis or encephalomyelitis, chronic meningitis, and stroke secondary to cerebral vasculitis (11–18).

A subset of individuals with Lyme borreliosis go on to experience persistent or relapsing-remitting symptoms including fatigue, pain, and neurocognitive difficulties after treatment; an illness referred to as post-treatment Lyme disease syndrome (PTLDS). These chronic symptoms are of sufficient severity to impact quality of life and physical functioning (15, 19–23). The current definition of PTLDS, developed by the Infectious Diseases Society of America (IDSA), is clinician-documented Lyme borreliosis treated with standard antibiotic regimens, with onset of fatigue, widespread musculoskeletal pain, or cognitive difficulties within 6 months of Lyme disease diagnosis and with continuous or relapsing symptoms persisting for at least 6 months after treatment has ended (24). Risk factors for the development of persistent symptoms include a delay in diagnosis and treatment, the severity of the initial infection, incomplete recovery at 4-months post-treatment, and a history of relapse (15, 21, 22, 25, 26).

While mounting scientific evidence in the last decade points to potential persistence of the bacterium *Borrelia* after antibiotic treatment, *in vitro* and *in vivo* (27–32), there exists ongoing confusion and controversy in the literature around PTLDS symptoms, including their etiology and management. The benefits of additional antibiotic therapy for PTLDS have been debated. Significant gains in certain domains have been reported in open label prospective studies utilizing extended antibiotic courses (33, 34), as well as two of the four randomized controlled trials of regimens containing intravenous ceftriaxone (20, 35–37); however, the authors of both randomized controlled trials that found gains in select domains with intravenous ceftriaxone therapy ultimately concluded that their studies did not support general use of IV ceftriaxone for PTLDS (35, 36). Although statistically significant improvements were seen in certain domains, their conclusions were based on risks of treatment as well as—in one trial—the lack of sustained benefit in cognitive improvement after completion of therapy (36), or—in the other—the benefit being limited to sustained improvement in a single domain, fatigue, which despite being a primary outcome measure, was deemed a “nonspecific” symptom (35). Issues surrounding the design of the randomized controlled trials and interpretation of their results have been debated (38–40). Importantly, studies to date have not led to comprehensive consensus guidelines for diagnosis and management of PTLDS. This underscores the need to more fully characterize its varied symptoms with the aim of better understanding potential underlying mechanisms which, in turn, can help inform management decisions.

While continuous or remitting fatigue, musculoskeletal pain, and/or cognitive difficulties are predominant, patients with persistent symptoms following LB treatment frequently report a variety of other symptoms including poor sleep, depression, visual disturbance, and sensory neuropathies that can be similarly burdensome and may affect fitness and function (41, 42). The cause of these persistent symptoms is not known, although several mechanisms have been proposed, including the direct neurotoxic effects of the spirochete, neuroinflammation, or autoimmunity (4, 43–48).

A symptom cluster is defined as a group of two or more symptoms that co-occur and are interrelated (49). The identification of symptom clusters has been used extensively in chronic conditions, including cancer (50, 51), inflammatory bowel disease (52), chronic obstructive pulmonary disease (53), and multiple sclerosis (54), in which symptoms seldom occur individually. Identifying the interrelationship between symptoms in a cluster can aid in more effective symptom management. For instance, symptoms may cluster together through a shared etiology such as neuroinflammation (55) or because they share the same triggering symptom (56). Identifying symptom clusters in individuals who report persistent symptoms following treatment for LB could aid in more effective symptom management through identifying triggering symptoms or an underlying etiology. The purpose of this study was to identify symptom clusters in individuals with persistent symptoms following treatment for LB and to examine the relationship between symptom severity and perceived disability in this population.

## Materials and Methods

### Cases

A retrospective chart review was conducted to examine symptoms and disability in individuals with a history of treatment for LB who were referred to The Dean Center for Tick-Borne Illness at Spaulding Rehabilitation Hospital in Boston. At the Dean Center, all patients completed symptom and disability surveys, which were incorporated into their medical chart. In addition, all patients underwent a complete blood count (CBC) and chemistry, tests of kidney, liver, thyroid function, and HgBA1c to rule out disorders that could mimic post-treatment Lyme borreliosis symptomatology (i.e., hypothyroidism, anemia, diabetes, etc.). Patients had serological testing for co-infections that are known or postulated to be tick-borne (i.e., Babesia, Anaplsama, Ehrlichia, Rickettsia, and Bartonella), either through their referring physician or at our center, and those with evidence of infection were treated according to established clinical protocols. The analysis of co-infection data have been omitted from this report and will be the subject of a separate study. Between 2015 and 2018, two-hundred and seventy adults (≥18 years) were identified by medical chart review. The Institutional Review Board approved this retrospective chart review, and data was de-identified prior to analysis. Responsible Conduct of Research (National Institutes of Health; Massachusetts General Hospital) and the Health Insurance Portability and Accountability Act of 1996 Privacy Rule were observed.

### Self-Report Symptoms and Functional Impairment

At the time of the first clinic encounter, each patient completed the 30-item General Symptom Questionnaire-30 (GSQ-30) which assesses symptom burden over the past 2 weeks (57) on a 0 to 4 scale where 0 = not at all, 1 = a little bit, 2 = somewhat, 3 = quite a bit, and 4 = very much. Patients also indicated whether any of the symptoms impaired their work, social, or family functioning, and if yes, which symptom was the most impairing. The GSQ-30 has shown excellent validity and internal consistency (57). The Sheehan Disability Scale (SDS) was also administered at the same time. The SDS is a widely used assessment of function in three domains: work/school, social life/leisure activities, and family life/home responsibilities (58). Each domain is scored using a 0–10 scale where 0 = not at all and 10 = extremely. The three domains are summed into a single-dimensional measure of global functional impairment with a range from 0 (no impairment) to 30 (highly impaired).

### Statistical Analyses

All statistical analyses were performed using IBM SPSS version 24.0 (IBM) and R version 3.6.1. Descriptive statistics and frequency distributions were calculated for demographic and clinical characteristics. We used the standard Cronbachs α coefficient to determine reliability. We used exploratory factor analysis (EFA) with principal axis factoring to identify factors or “symptom clusters.” The key concept of EFA is that multiple items on the GSQ have similar patterns of responses across individuals because they are all associated with a latent (i.e., not directly measured) variable. Principal axis factoring with oblique rotation (Varimax) was used as the factor model with squared multiple correlations used to establish communalities. The Kaiser-Meyer-Olkin test, a measure of how suited our dataset was for EFA, verified the sampling adequacy for the analysis; KMO = 0.922. The number of factors was determined using a scree plot and the total percentage of variance explained by each factor with an eigenvalue greater than the average eigenvalue. A factor loading ≥0.4 was used to identify significant factors, with at least two items loaded in each cluster (59). As symptoms are complex and could be cross-loaded on more than one factor, the decision to retain the symptom on one factor was based on the significance of the loading and the conceptual and clinical relevance of the symptom. Each factor orsymptom cluster was assigned a name to reflect the possible underlying etiology. Three items were removed from the EFA due to insufficient variation in the occurrence of these symptoms: shortness of breath, feeling feverish, and sweats, and/or chills.

## Results

Two-hundred and seventy adult cases were identified, of which 67.8% were female, with a mean age of 49 ± 14.8 years (Range 18–88) and 16.1 ± 1.4 years of education. For employment status, 12.7% were on disability or unemployed, 8.5% were retired, 6.8% were students, 2.5% were homemakers, and 69.6% were employed. The mean time since LB diagnosis and treatment was 10 ± 8.2 years (Range 1–43 years, median = 8 years).

Table 1 shows the mean symptom severity scores and symptom rankings based on responses to each of the 30 questions on the GSQ-30. Potential scores ranged from 0 (Not at all) to 4 (Very much). The mean symptom severity score range for the total study population was 0.7 ± 1.1 for “*feeling feverish”* to 2.9 ± 1.3 for “*feeling fatigued or having low energy*.” Over 80% (*n* = 220) of patients reported “yes” when asked whether any of the symptoms impaired work, social or family function. The top five symptoms identified as the greatest cause of impaired work, social, or family function, making up over 50% of respondents, were feeling fatigued or having low energy, slower speed of thinking, muscle aches or pains, joint pain or swelling, and trouble with memory (Table 1).

**Table 1:** Symptom severity and impairment scores.

**Symptom^**a**^**	**Rank**	**Severity**^**b**^		**Impaired^**c**^**
		**Mean**	**SD**	**%**
Feeling fatigued or having low energy	1	2.9	1.3	20
Muscle aches and pains	2	2.5	1.3	7.9
Not feeling rested upon wakening	3	2.5	1.5	1.4
Trouble with memory	4	2.4	1.3	6.0
Feeling worse after normal physical exertion	5	2.4	1.5	2.3
Slower speed of thinking	6	2.4	1.4	10.2
Trouble finding words or retrieving names	7	2.3	1.4	4.7
Needing more sleep than usual	8	2.2	1.5	1.4
Trouble falling or staying asleep	9	2.2	1.5	2.3
Joint pain or swelling	10	2.2	1.5	6.5
Stiff or painful neck	11	2.1	1.5	1.9
Muscle weakness	12	2.1	1.4	2.8
Back pain	13	2.1	1.5	6.0
Numbness or tingling	14	1.9	1.5	0.9
Headaches	15	1.9	1.4	5.1
Feeling irritable, sad, or decreased pleasure	16	1.9	1.4	2.8
Feeling panicky, anxious, or worried	17	1.8	1.5	3.7
Shooting, stabbing or burning pains	18	1.8	1.5	0.9
Change in visual clarity or trouble focusing	19	1.8	1.5	0.9
Discomfort with normal light or sound	20	1.7	1.5	2.3
Balance problems or sense of room spinning	21	1.7	1.5	2.3
Skin or muscle twitching	22	1.6	1.5	0.9
Hot or cold sensations in extremities	23	1.6	1.5	0.5
Light headed or uncomfortable on standing	24	1.6	1.4	0.5
Sweats and/or chills	25	1.4	1.3	1.9
Bladder discomfort or change in urination	26	1.2	1.4	-
Irregular or rapid heart beats	27	1.1	1.3	0.9
Nausea and/or vomiting	28	1.1	1.3	1.4
Shortness of breath	29	1.0	1.1	1.4
Feeling feverish	30	0.7	1.1	-

*SD is standard deviation*.

a *Patients were asked how much they had been bothered by each of the symptoms listed during the past 2 weeks*.

b *Symptom severity: 0 = not at all, 1 = a little bit, 2 = somewhat, 3 = quite a bit, 4 = very much*.

c *% of total respondents (N = 215) who identified symptom as most impairing on work, social, or family functioning*.

**Figure 1:**
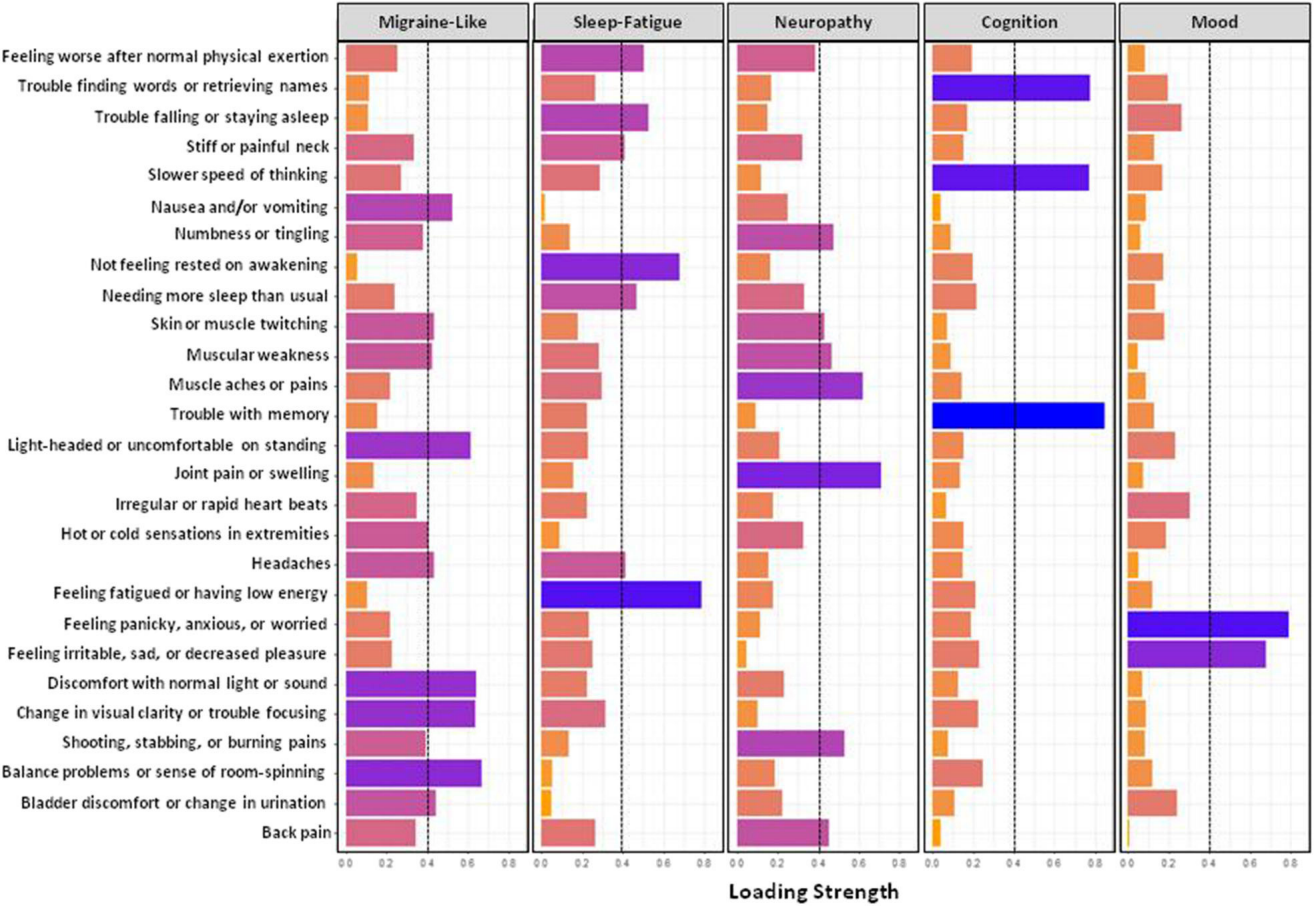
Exploratory factor analysis using scores on the GSQ-30.

**Figure 2:**
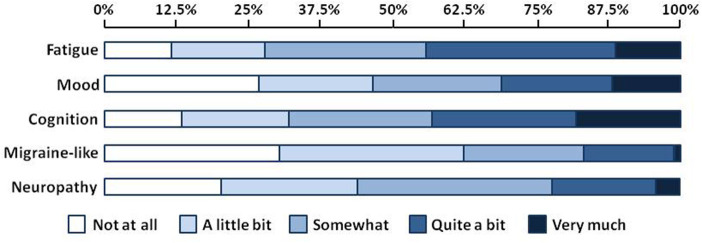
% of patients reporting being bothered by symptoms in each symptoms clusters.

Figure 1 shows results from the exploratory factor analysis for responses on the GSQ-30 symptom survey in all patients. The six items in factor 1 (balance problems, discomfort with normal light and sound, nausea and/or vomiting, etc.) were called the migraine-like symptom cluster. The six items in factor 2 (feeling fatigued or having low energy, needing more sleep than usual, etc.) were called the sleep-fatigue symptom cluster. The eight symptoms in Factor 3 (i.e., muscle aches and pain, numbness and tingling, shooting, stabbing and burning pains, etc.) were called the neuropathy symptom cluster. The three symptoms in factor 4- trouble with memory, slower speed of thinking, and trouble finding words or retrieving names- were called the cognitive symptom cluster. Finally, the two items in factor 5, feeling panicky, anxious, or worried and feeling irritable, sad, or decreased pleasure, were called the mood symptom cluster. Figure 2 shows the percentage of patients who reported being bothered by symptoms in each of the five symptom clusters ranging from “not at all” to “very much.” Approximately 45% of patients reported that they were troubled quite a bit or very much by fatigue or cognitive difficulties. Mood symptoms were the next most troubling, with approximately 30% of patients reporting that they were bothered quite a bit of very much by these symptoms. Although migraine-like and neuropathic symptoms were the least troublesome, they were still troubling for approximately 20% of patients.

**Figure 3:**
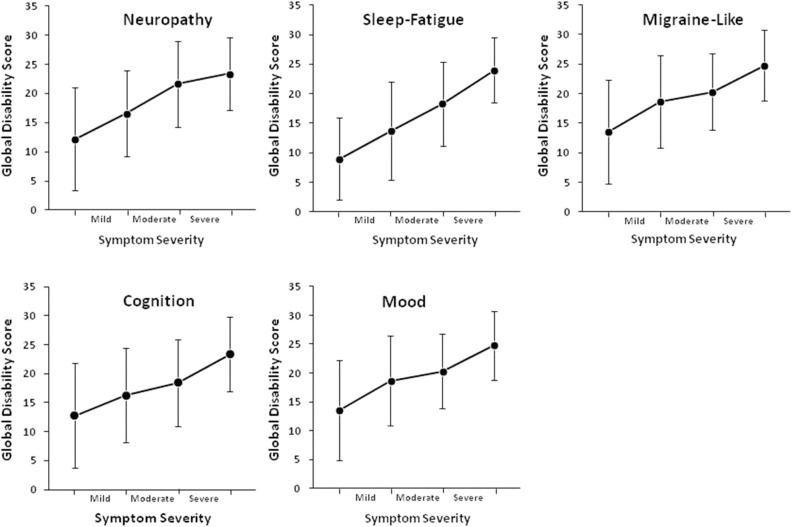
Effect of increasing symptoms severity of functional impairment.

SDS data was available for 220 patients. Mean scores on the SDS work/school, social life/ leisure activities, and family life/home responsibilities domains were 5.7 ± 3.5, 6.5 ± 3.0, and 6.2 ± 3.1, respectively. The mean Global Functional Impairment score was 18.2 ± 8.9. Increasing symptom severity for each symptom cluster was linearly associated with greater global disability (*p* < 0.001, Table 2). Functional impairment increased when the severity of fatigue, cognitive, mood, and migraine-like symptoms increased from moderate to severe, and when neuropathy symptoms increased from mild to moderate (Figure 3).

**Table 2:** Associations between symptom severity and global disability score.

**Symptom cluster**	**β Coefficient (95% CI)**
Neuropathy	0.46 (2.59–4.38)^*^
Fatigue-Sleep	0.57 (3.43–5.05)^*^
Migraine-like	0.54 (3.43–5.22)^*^
Cognition	0.44 (2.10–3.68)^*^
Mood	0.44 (2.28–3.73)^*^

* *p < 0.001*.

## Discussion

This is the first study to examine symptom clusters in a large cohort of individuals with persistent symptoms following treatment for Lyme borreliosis. The identification of symptom clusters may help us to identify mechanisms, and allow us to correlate clusters to specific infectious agents. We identified five symptom clusters, which we named the neuropathy, sleep-fatigue, migraine-like, cognitive, and mood symptom clusters. The sleep-fatigue symptom cluster included three items related to sleep quality: needing more sleep than usual, not feeling rested upon awakening, and trouble falling or staying asleep. Prior studies have reported poorer sleep in individuals with PTLDS compared to healthy controls (41, 60, 61), and self-reported fatigue and perceived poor sleep quality frequently co-occur in other chronic conditions, including cancer (62), diabetes (60, 63), and chronic fatigue syndrome (64). While there are frequent reports of sleep disruption in LB, only one study has examined both patient-reported and objectively measured sleep outcomes in this population. Greenberg et al. compared self-reported and polysomnographic assessment of sleep quality in LB patients and in matched healthy controls (65). Compared to healthy controls, LB patients reported greater difficulty falling asleep, more restless sleep, and increased daytime sleepiness (65). Objective sleep assessment revealed an increase in onset latency (time to fall asleep), decreased sleep efficiency (time in actual sleep divided by time attempting to sleep), and higher frequency of awakenings (65). Poor sleep quality could be the triggering symptom in this cluster since inadequate sleep would likely increase fatigue. Alternatively, individuals with high levels of fatigue, in particular, daytime sleepiness, could resort to daytime napping, which, in turn, could lead to fragmented and non-refreshing sleep at night. Further characterization of sleep deficits in people with persistent symptoms post LB are needed to develop strategies aimed at improving sleep and possibly fatigue in LB patients with documented sleep deficits.

The majority of the symptoms in the neuropathy symptom cluster were related to musculoskeletal pain and weakness, paresthesia, and hot and cold sensation in the extremities, symptoms common in small fiber neuropathy (SFN). In a recent study, Novak et al. examined SFN in individuals with persistent LB symptoms and found abnormal epidermal nerve fiber density (ENFD) in 90%, abnormal sweat gland nerve fiber density (SGNFD) in 50%, and both ENFD and SGNFD in 40% (42). Consistent with these sensorimotor deficits, many LB patients have been shown to have a reduced vibration threshold in their extremities. In the study by Rebman et al., approximately 30% of LB patients had scores below the age-adjusted cutoffs for vibration threshold in upper and lower extremities (41). Despite evidence of somatosensory deficits in the lower extremities, there have been no studies to date that have performed a detailed examination of gait and balance deficits in LB. Although the mechanism whereby LB causes SFN is not known, several mechanisms of neuronal injury have been proposed, including the direct neurotoxic effects of the spirochete, neuroinflammation, or autoimmunity (4, 43, 46, 48).

Neuroinflammatory processes may also underlie the symptoms found in the migraine symptom cluster, which included items related to visual disturbances, sensitivity to light or sound, balance problems, or being lightheaded or uncomfortable while standing. Indeed, we labeled this symptom cluster the migraine-like cluster because these symptoms are common in migraine. The overlap between migraine symptoms and those of post-treatment LB suggests that they may share a common mechanism. Migraine is a chronic neurological disorder that affects the central and peripheral nervous systems (66). Altered activity of the thalamic and thalamo-cortical areas contribute to aberrant sensory processing inherent in migraine, while MRI studies have demonstrated altered connectivity in a number of brain regions, including the cerebellum, hypothalamus, and brain stem (66). Recently the neurogenic inflammatory mediator calcitonin gene-related peptide (CGRP) has been implicated in the etiology of migraine (67). CGRP is produced by neurons in the CNS and the peripheral nervous system, where it acts as a vasodilator and inflammatory mediator acting via NF-κB (68–70). CGRP is released from neurons in response to a variety of environmental stimuli, including infectious agents such as *B. burgdorferi* (71). Consistent with its role in the etiology of migraine, individuals with acute migraine have elevated circulating levels of CGRP (72), and newly developed therapeutic monoclonal antibodies that inactivate circulating CGRP have proven efficacy in the treatment and prevention of migraine (67). Whether the same tools used to understand the cause of migraine could be applied to identify the cause of migraine-like symptoms in LB remains to be seen but could be a fruitful avenue for future investigation.

Items in the cognition symptom cluster included trouble with memory, trouble finding words or retrieving names, and slower speed of thinking. Self-reported cognitive deficits are frequently reported in LB and in other neurological and inflammatory conditions, including cancer, rheumatoid arthritis, and multiple sclerosis. However, self-reported cognitive deficits do not always correlate with performance on neuropsychological tests. Berende et al. found no association between self-reported cognitive difficulties in over two hundred LB patients and performance on objective tests of episodic memory, working memory / attention, verbal fluency, information-processing speed, and executive function (73). Less than 3% of participants had cognitive deficits based on neuropsychological testing, a rate comparable to the general population. Their findings were similar to the study by Kaplan et al. who similarly found no association between subjective cognitive difficulties and performance on tests of memory, attention, and executive functioning in 129 individuals with physician documented LB (74). Touradji et al. found that while over 90% of 124 LB patients reported cognitive difficulties, only 26% showed evidence of mild cognitive deficits in memory and processing speed (75). In contrast, Tager et al. reported significantly more objective cognitive deficits and psychiatric disturbances in children who developed new-onset cognitive complaints after Lyme disease compared with matched healthy controls (76). Objective cognitive deficits on neuropsychological evaluation, which included disturbances in visual and auditory processing and attention as well as in working memory and mental tracking, were still found after controlling for anxiety, depression and fatigue (76). Similarly, Keilp et al. observed statistically significant differences in several cognitive tests including tests of verbal comprehension, attention, executive function, working memory, and processing speed between patients with a history of LB and healthy controls (77). Like the Touradji study, Keilp et al. noted that the observed cognitive deficits were mild. Discordance between self-report and objectively measured cognitive function is not unique to LB and may reflect limitations in current neuropsychological testing which do not incorporate “real-world” demands on cognitive function. In the real world setting, cognitive tasks are frequently performed concurrently with motor tasks (i.e., walking while talking). While dual-tasking (i.e., performing cognitive and motor tasks concurrently) poses little problem for people with intact cognitive and sensorimotor function, it can be problematic for individuals with cognitive or motor deficits such as older adults and those with neurological conditions such as diabetes, stroke, or multiple sclerosis in which balance and cognitive deficits frequently co-exist (78–82). Given the reported sensorimotor deficits in LB patients, testing performance on cognitive tasks with a concurrent motor task may be a better indicator of cognitive decline than performance on a cognitive task administered alone under laboratory conditions. Further work is needed to fully understand the scope of neurocognitive problems in LB patients and to identify neural pathways that contribute to these deficits.

Finally, the two items in the mood symptom cluster- feeling panicky, anxious, or worried and feeling irritable, sad, or having decreased pleasure- are common symptoms of anxiety and depression. Symptoms of irritability and depression have been documented in LB (83–89) although it is unclear whether these symptoms are of sufficient severity to meet criteria for clinical anxiety/depression (89). A prior population-based retrospective cohort study did not show increased rates of depression in individuals with a history of LB and persistent symptoms compared to those without symptoms (19).

There are several limitations to the current study, including its retrospective, cross-sectional design. Data used in the analyses were extracted from the medical record and therefore lacked consistently documented clinical and demographic information that would typically be collected in a prospective research study. Although all patients seen at the clinic had a history of treatment for LB and were referred because of lingering symptoms, their charts lacked several pieces of information needed to determine whether they met the criteria for a diagnosis of PTLDS. Future studies should include a detailed analysis of the infectious origins of symptoms in these patients (i.e., multiplex PCR analysis combined with serology), which would allow us to determine whether specific clusters correlate to a particular infectious organism, or allow differential diagnoses. Because only clinical data from the initial clinic visit was used to create the symptom clusters, the stability of these clusters over time is not known. Future studies are needed to identify shared or distinct mechanisms, including distinct infectious organisms, that underlie these symptom clusters, which will aid in the development of new treatment strategies.

## Data Availability Statement

The datasets generated for this study are available on request to the corresponding author.

## Ethics Statement

The studies involving human participants were reviewed and approved by Spaulding Rehabilitation Hospital Review Board. Written informed consent for participation was not required for this study in accordance with the national legislation and the institutional requirements.

## Author Contributions

NZ, CM, EB, and LW had full access to all the data in the study and take responsibility for the integrity of the data and the accuracy of the data analysis. LW and EB conducted the statistical analyses. LW and NZ take responsibility for the study's concept and design. LW, NZ, CM, and EB participated in the drafting of the manuscript. NZ and DC obtained funding. QW and RO provided administrative, technical, or material support. All authors contributed to the acquisition, analysis or interpretation of the data and all contributed to critical revision of the manuscript for important intellectual content.

## Conflict of Interest

The authors declare that the research was conducted in the absence of any commercial or financial relationships that could be construed as a potential conflict of interest.
